# Multimodal Prediction of Five-Year Breast Cancer Recurrence in Women Who Receive Neoadjuvant Chemotherapy

**DOI:** 10.3390/cancers14163848

**Published:** 2022-08-09

**Authors:** Simona Rabinovici-Cohen, Xosé M. Fernández, Beatriz Grandal Rejo, Efrat Hexter, Oliver Hijano Cubelos, Juha Pajula, Harri Pölönen, Fabien Reyal, Michal Rosen-Zvi

**Affiliations:** 1 IBM Research-Israel, Mount Carmel, Haifa 3498825, Israel; 2 Institut Curie, 26 Rue d’Ulm, 75005 Paris, France; 3 VTT Technical Research Centre of Finland, Kivimiehentie 3, 02150 Espoo, Finland; 4 Faculty of Medicine, The Hebrew University, Jerusalem 91120, Israel

**Keywords:** breast cancer recurrence, neoadjuvant chemotherapy, magnetic resonance imaging (MRI), machine learning, deep learning, image processing, radiomics

## Abstract

**Simple Summary:**

An important clinical issue to consider when selecting neoadjuvant chemotherapy treatment for breast cancer is the likelihood of cancer recurrence. Accurately predicting the future outcome of the patient based on data available prior to treatment initiation could impact the treatment selection. We study a cohort of 1738 patients and explore the contribution of clinical history, immunohistochemical markers, and multiparametric magnetic resonance imaging to the prediction of post-treatment cancer recurrence. We analyzed this multimodal data using classical machine learning, image processing, and deep learning to increase the set of discriminating features. Our results demonstrate the ability to predict recurrence using each modality alone, and the possible improvement achieved by combining the modalities. We show that the models are especially accurate for differentiating specific groups of young women with poor prognoses. These methods were also used on a different dataset of 193 patients in an international challenge, where they won second place.

**Abstract:**

In current clinical practice, it is difficult to predict whether a patient receiving neoadjuvant chemotherapy (NAC) for breast cancer is likely to encounter recurrence after treatment and have the cancer recur locally in the breast or in other areas of the body. We explore the use of clinical history, immunohistochemical markers, and multiparametric magnetic resonance imaging (DCE, ADC, Dixon) to predict the risk of post-treatment recurrence within five years. We performed a retrospective study on a cohort of 1738 patients from Institut Curie and analyzed the data using classical machine learning, image processing, and deep learning. Our results demonstrate the ability to predict recurrence prior to NAC treatment initiation using each modality alone, and the possible improvement achieved by combining the modalities. When evaluated on holdout data, the multimodal model achieved an AUC of 0.75 (CI: 0.70, 0.80) and 0.57 specificity at 0.90 sensitivity. We then stratified the data based on known prognostic biomarkers. We found that our models can provide accurate recurrence predictions (AUC > 0.89) for specific groups of women under 50 years old with poor prognoses. A version of our method won second place at the BMMR2 Challenge, with a very small margin from being first, and was a standout from the other challenge entries.

## 1. Introduction

Breast cancer remains the most widely diagnosed cancer and the leading cause of death among women today [1]. One of the options for treating locally advanced breast cancer is neoadjuvant chemotherapy (NAC), in which chemotherapy and optionally targeted treatment are administered prior to surgery. Potential clinical advantages of NAC have been largely studied and include improving the rate of breast-conserving therapy, obtaining accurate in vivo tumor sensitivity, and a correlation between the response to primary chemotherapy and overall survival [2].

The decision to select NAC or an alternative treatment is mainly based on well-established prognostic biomarkers or factors. A prognostic biomarker/factor is any measurement available before treatment that correlates with disease-free or overall survival in the absence of systemic adjuvant therapy and, as a result, can relate to the natural history of the disease. Several breast cancer prognostic factors have been discussed in the literature, including clinical, pathological, and biological parameters. In our setting, the well-established prognostic biomarkers/factors include age, hormone receptor status, human epidermal growth factor receptor 2 (HER2), tumor size, number of affected nodes, disease grade, tumor proliferation rate, clinical stage, and lymphovascular involvement [3]. Typically, tumor location and size are estimated from medical images and used in daily clinical practice, but no other radiographic information is used for treatment selection.

An important clinical issue to consider when selecting NAC treatment is the likelihood of future cancer recurrence or determining if the cancer is likely to recur either locally in the breast (local relapse) or in other distant areas in the body (metastasis). Accurately predicting the future outcome of the patient based on data available prior to the initiation of NAC could inform the therapeutic options and impact the treatment selection. Today, aside from the use of the above-mentioned prognostic biomarkers and factors, clinicians are limited in their ability to assess which of the NAC-treated patients will suffer a recurrence. In fact, the progression of the disease of a group of patients characterized by similar prognostic biomarkers is often nonunanimous. Some patients are recurrence-free, while others encounter cancer recurrence. For this reason, newer prognostic biomarkers and factors are needed to help perform precision medicine in an attempt to define disease-related prognosis more accurately. The study described in this paper holds the promise of identifying novel composite prognostic biomarkers that would enable more accurate outcome prediction. Clinicians’ treatment selection and decision-making could be assisted and empowered by artificial intelligence models that could accurately predict recurrence. Moreover, discovery of novel composite biomarkers could lead to a better understanding of the heterogeneous nature of this cancer [4].

In this paper, we present multimodal AI models that predict cancer recurrence within five years from diagnosis, using both clinical data and multiparametric magnetic resonance imaging (mpMRI) taken prior to treatment. Predicting treatment outcome using mpMRI is an emerging area of interest in the medical community [5] and an important enabler of precision medicine. Our study examines the contribution of mpMRI in the case of breast cancer. We combine a deep learning model and automatic image processing and radiomics techniques for mpMRI, a classical machine learning model for clinical data, and an ensemble model of the individual clinical and mpMRI models. We evaluate and compare the models using the receiver operating characteristic (ROC) curve and the area under the ROC curve (AUC) with confidence interval. We then use several metrics to evaluate and compare the models at high-sensitivity operation points and the statistical significance (*p*-value) of this comparison. High-sensitivity operation points are used in models deployed in clinical practice and thus are of special interest. We also use interpretability methods [6] to explain the model and identify important clinical features for predicting recurrence that combined together can serve as novel candidate composite biomarkers. Finally, we analyze several subgroups of patients experiencing similar prognostic biomarkers. We show that our model can improve the discrimination within the subgroup between the recurrence-free patients and those that will encounter recurrence.

The rest of the paper is organized as follows: We present the methods used to develop our multimodal predictor in Section 2 and the evaluation of our models in Section 3. We describe the work related to this topic in Section 4. We then discuss our results in Section 5 and finalize with conclusions in Section 6.

## 2. Materials and Methods

We worked with a real-world retrospective dataset of patients, composed entirely of women diagnosed with breast cancer who received NAC. The data of each patient include clinical information such as height, weight, age, histological type of the tumor, progesterone status, and many more features. We consider all these data as a single clinical modality. Some of the patients also had in their record the MRI data acquired prior to NAC initiation, which are considered a second modality. Given that we have different sizes of datasets for the different modalities, we divided our model into two branches. One branch was trained using clinical data and images, while the other branch was trained using only clinical data. We then combined the two branches into one final ensemble model. In this section, we present the study design and our dataset, describe the mpMRI model branch and the clinical model branch, and then detail the final ensemble model that combines the two branches.

### 2.1. Study Design

**Figure 1:**
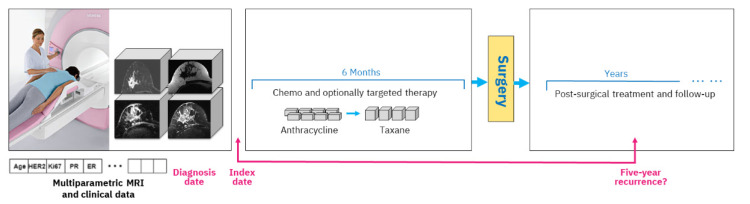
Study setting. Multiparametric MRI and clinical data acquired prior to NAC treatment start are analyzed to predict breast cancer recurrence within five years after diagnosis. The NAC treatment includes six months of chemotherapy with optional targeted treatment followed by surgery. After surgery, there is follow-up and sometimes additional treatment such as radiotherapy.

A typical study design includes an index date, and in our case, we use the date of the mpMRI exam acquired prior to treatment start. All data that are available up until that date are considered clinical history, including various clinical tests and immunohistochemical markers. The diagnosis date on which the cancer was diagnosed by biopsy is generally very close to the mpMRI acquisition date (up to six weeks difference). During treatment, the woman obtains chemotherapy with optional targeted medication, followed by surgery and post-surgical treatment. Outcome assessment is established based on five-year recurrence counted from the diagnosis date, which is approximately equivalent to the index date. Figure 1 depicts the overall study setting.

The mpMRI imaging is routinely carried out prior to NAC initiation. Our study includes four types of MRI imaging. The first type, dynamic contrast-enhanced MRI (DCE-MRI) volumes, acquires T1 changes in tissues before and after the injection of a gadolinium-based contrast agent. We used subtraction volumes that are digital subtraction between the DCE-MRI volume acquired after the injection of the contrast agent and the baseline volume acquired before the injection. The second type of imaging that we used is apparent diffusion coefficient (ADC) volumes, which are derived from a diffusion-weighted MRI (DW-MRI) imaging series. The third and fourth types of MRI volumes are water and fat suppression volumes from the Dixon multi-echo MRI series that enable, respectively, the visibility of fat or water in the breast tissue.

### 2.2. Dataset

Our dataset includes a collection of 1738 patients that received NAC treatment between 2012 and 2018 at Institut Curie in France. In this collection, 11 patients had metastasis disease already at the time of diagnosis and hence were excluded from the data, resulting in 1727 patients.

A complete description of the dataset characteristics can be found in Appendix A. For each patient, the data describe whether the patient encountered relapse or metastasis after treatment, the number of days from birth to diagnosis, and then the number of days to relapse or metastasis if they occurred, among other information. In addition, the dataset includes a binary flag indicating whether the patients are censored or not, i.e., if they were diagnosed within the last five years. Since the anonymization process applied to the hospital health records did not allow us to have exact dates, we could not use regression models such as the Cox [7] model to handle the censored patients. As a result, we utilized a different approach.

A randomly selected subset of the cohort of patients who had clinical and MRI data, 100 patients, was set aside for holdout evaluation. The remaining 1627 patients were considered for our training cross-validation experiments. The distribution of positive recurrence patients in training and holdout was approximately 16% in each. The censored data in the holdout cohort were excluded from evaluation, resulting in 62 uncensored patients.

Table 1 summarizes the cohorts in our dataset. Because some patients had only clinical data while other patients had both clinical and MRI data, we used two training cohorts in our experiments. The first cohort, referred to as the Clinical cohort, is the full cross-validation dataset of 1627 patients for clinical data evaluation. The clinical data included patients’ characteristics such as age, weight, and height; tumor properties such as breast cancer histology, tumor grade, and Ki67; and immunohistochemical subtypes based on estrogen, progesterone, and HER2 expression. This cohort consisted of 928 uncensored patients diagnosed 5 years or more before data collection, and 699 censored patients diagnosed less than 5 years before data collection.

The second cohort, the MRI+Clinical cohort, includes 463 patients who had MRI scans taken prior to NAC treatment, in addition to their clinical data. The MRI scans had several types of series taken in the same visit, including DCE-MRI volumes acquiring contrast agent effects, ADC volumes derived from diffusion-weighted imaging, and Dixon volumes with both water suppression and fat suppression. This cohort included 317 uncensored patients, and 146 censored patients that were diagnosed less than 5 years before data collection. The MRI+Clinical cohort is a subset of the Clinical cohort.

### 2.3. Multiparametric MRI Model

We split the MRI+Clinical cohort of 463 patients with clinical and MRI data into 5 folds and performed 5-fold cross-validation. In that process, we iteratively selected a different validation fold and trained on the remaining 4 folds, resulting in 5 models validated on different folds. For the holdout evaluation, the patient score is the average of the scores of the five models selected from the 5-fold cross-validation.

Our approach to handling censored patients was to exclude them from the respective validation fold but keep them in the other folds that served for training. The label of the censored patients when used in training was the prediction obtained from a side model. The side model was created by training a random forest classifier on the uncensored patients. This approach allowed us to retain the amount of training data while having an accurate validation evaluation as our evaluation was performed only on uncensored patients.

**Figure 2:**
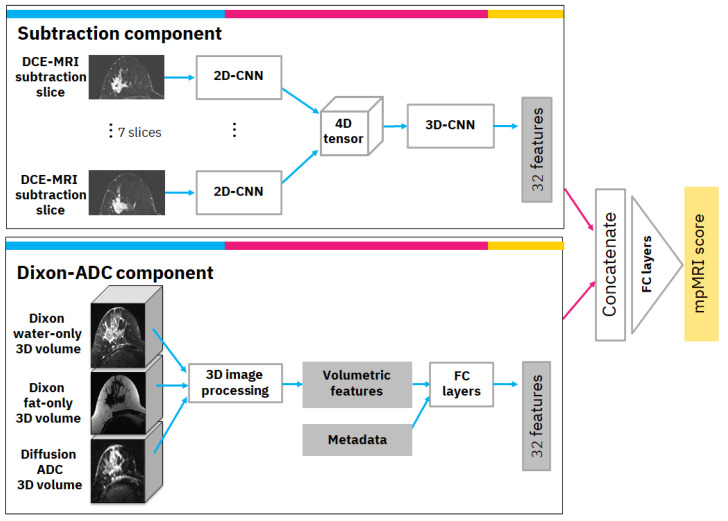
Multiparametric MRI model architecture. (**Top**) Subtraction component in which seven adjacent MRI slices (three pre-significant, significant, three post-significant) form the input to seven 2D-CNNs that have the same weights. The features are aggregated into a 3D-CNN followed by an average global pooling layer. (**Bottom**) Dixon-ADC component in which three 3D MRI volumes form the input to volumetric 3D image processing that generates volumetric features. The features from the two components are concatenated and transformed into the output score.

The mpMRI model uses multiple volumes of the same study and consists of two components. The first is the subtraction component with a convolutional neural network (CNN) model that receives annotated subtraction volumes as input and produces 32 deep features. The annotation includes the most important subtraction volume, in which the tumor appears to be the brightest in terms of relative illumination. In the selected volume, the annotation also includes the most significant slice in which the tumor was the largest. The second component is the Dixon-ADC component, which receives a Dixon series consisting of fat-only and water-only volumes as input, as well as a series of diffusion-weighted MRI-derived ADC volumes. Image processing and radiomics methods are used to generate morphological and texture volumetric features that are augmented with clinical metadata. Finally, the features from both components are concatenated and transformed to produce the mpMRI model score. A detailed diagram of the mpMRI model architecture is depicted in Figure 2. The following subsections describe each component and how it is integrated into the overall mpMRI model.

#### 2.3.1. Subtraction Component

The subtraction component is based on a CNN from previous work [8]. The input to the CNN is the significant slice and the six pre- and post-adjacent slices (i.e., seven slices in total) that are extracted from the selected DCE-MRI subtraction volume. The selected slices undergo a cropping and resizing process as follows: First, since our data consisted of axial MRI volumes that contain both sides of the breast, we cropped the image vertically and continued processing only the relevant side in which the tumor was located. Next, we cropped the image horizontally to exclude non-breast parts that appeared in the image. Each of the vertically and horizontally cropped slices was then resized to 512 × 256 pixels to bring them all to the same size. The last two steps of the preprocessing included rotating the slices, so the breast was facing in the same direction for all slices, and under-sampling the slices where there was overlap between slices in the volume.

Our CNN model is a modification of ResNet [9] as a classifier. We specifically used the ResNet18 formulation but reduced the number of filters per layer to speed up training and avoid over-fitting. For our network, we used 7 residual blocks with (32, 64, 64, 128, 128, 256, 256) filters per convolutional layer. This 2D-CNN model was applied simultaneously to the 7 slices; i.e., the same 2D-CNN model with the same weights was applied to each slice. Next, we used a 4D-tensor to aggregate features produced from the 7 input slices. Finally, a 3D convolution layer was applied, followed by a 3D average global pooling layer. The output of the pooling layer includes 32 features that are the output of this component.

#### 2.3.2. Dixon-ADC Component

The Dixon-ADC component is based on fuzzy c-means (FCM) clustering for automatic image segmentation with two phases. The first phase uses FCM to segment the whole MRI volume and detect voxels that belong to the breast tissue. The approach follows Klifa et al. [10], but we use water-only and fat-only Dixon volumes. In the second phase, voxels are once again clustered with FCM, but this time to segment the lesions. This phase also uses the diffusion ADC volume when available. The clusters with the biggest overlap on high-intensity areas on ADC and water-only volumes are selected as lesion regions. This process generates three 3D masks: bilateral breast tissue mask, tumor side breast tissue mask, and lesion mask. An example of this segmentation can be found in Appendix B.

Based on the automatically generated 3D masks, various imaging features describing the morphological properties of the lesions and the breast tissue were generated; these include tumor size, volume, shape, intensity, and texture under different masks and MRI sequences. If the segmentation algorithm had produced multiple separate lesion volume masks, only the largest volume was used to calculate the volumetric features. The most important features were found to be: (1) tumor volume in cubic millimeters; (2) tumor surface area in square millimeters; (3) number of separate regions in tumor segmentation; (4) mean intensity of tumor region in comparison to other breast tissue intensity using water suppression volume of Dixon MRI sequence; and (5) spread of the tumor: the sum of the maximum lengths of the x, y, and z dimensions of the tumor in the 3D volume, in millimeters. In practice, any of these designed features can be visualized for the clinical operator with MRI volume if necessary. Finally, the volumetric features were augmented with clinical metadata and transformed via 5 fully connected layers with (16, 16, 16, 32, 32) filters to 32 features.

#### 2.3.3. Overall mpMRI Model

The overall mpMRI model is based on DCE-MRI subtraction and Dixon and ADC volumes of the same study. The computed DCE-MRI subtraction deep features and the computed Dixon-ADC volumetric-based features are concatenated and transformed via fully connected layers with (64, 32, 1) filters to produce the mpMRI score.

We performed 5-fold cross-validation and computed the ROC AUC with confidence interval (CI). We selected several high-sensitivity operation points that are of clinical interest and computed their specificity and other metrics. We also used the model to evaluate the holdout data ROC AUC and various metrics at the chosen high-sensitivity operation points.

### 2.4. Clinical Model

We split the Clinical cohort of 1627 patients with clinical information into 5 folds with equally distributed positive and negative samples among folds. The folds were created in correlation with the folds of the MRI+Clinical cohort; i.e., a patient remains in the same fold in both cohorts. In addition, similar to the MRI model, in each iteration of the cross-validation, the censored patients were excluded from the fold that was used for validation but were retained in the other folds that were used for training.

To select the best classifier for our task, we preprocessed and trained the data with three known machine learning algorithms: random forest, logistic regression, and XGBoost. The preprocessing included a scaler that scaled all features to the [0, 1] range and an imputation process to replace missing values with the mean value. Since our data were highly unbalanced, we used sample weighting that is inversely proportional to the class frequencies in the input data for the random forest and logistic regression classifiers. For XGBoost, we used positive scaling that is proportional to the ratio between negative and positive samples.

We performed cross-validation and computed the ROC AUC with CI. We selected several high-sensitivity operation points that are of clinical interest and computed specificity and other metrics for them. We then selected the best model, which ended up being random forest. We used that model to evaluate the holdout data AUC and metrics at the selected high-sensitivity operation points. We also examined the features of importance produced by our clinical model using the Shapley Additive Explanations (SHAP) algorithm [6], an interpretability method that demonstrated how each feature of each patient affects the predictive model results.

### 2.5. Ensemble Model and Subgroup Analysis

The ensemble model receives six scores per patient: three scores based on clinical data and three scores based on the MRI data. To improve generalization, we created multiple variations of each model using a method similar to [8], in which a different variation started its training with a different seed. Thus, the three scores for clinical data are produced from three clinical models’ variations that differ in their training seed initialization, and the three scores for MRI data are produced from three MRI models’ variations. Each clinical or MRI model is calibrated using Platt’s method [11] to normalize the scores of each model. We then examined several strategies for combining and ‘ensembling’ the models. However, we found that the most effective strategy used the mean value of all available scores per patient.

The final ensemble model and the scores it obtained for the validation and holdout sets for the uncensored patients were the basis for our subgroup analysis. Based on clinicians’ suggestions, we divided our MRI+Clinical cohort and the Clinical cohort based on the values of age, cancer subtype, histological type, tumor grade, and Ki67. We evaluated the AUC with CI for each subgroup and explored whether matching patients with similar prognostic parameters can be differentiated by our model.

## 3. Results

We evaluated the individual per modality models and the ensemble model. The final ensemble model was evaluated on the MRI+Clinical cohort since it included both MRI and clinical data, and we could compare the contribution of each modality. As part of our evaluation, we performed a 5-fold cross-validation as well as a test on a holdout dataset. For both the cross-validation and holdout independent test, we report AUC with a 95% CI, and for several clinically important high-sensitivity operation points, we report specificity, F1-score, balanced accuracy, positive predictive value (PPV), and negative predictive value (NPV). We also explain the model using the SHAP algorithm to identify important clinical features. Finally, we report AUC with 95% CI on several subgroups exhibiting similar prognostic biomarkers.

### 3.1. Model Evaluation

Table 2 summarizes the results of the cross-validation and holdout independent test. When using just the subtraction component without using the Dixon-ADC component (row 1), we achieved in cross-validation lower results than when using the mpMRI that combines both the subtraction and Dixon-ADC MRI volumes (row 2). In the clinical model branch (row 3), the cross-validation obtained similar AUC to the mpMRI but on a completely different modality.

The mpMRI model shows an advantage over the subtraction-only model also in the ensemble model. The ensemble of the subtraction-only MRI with the clinical model (row 4) achieved lower results than using the mpMRI for the ensemble. The mpMRI and clinical ensemble (row 5) received the best results and was thus selected as the final model. The final model achieved an AUC of 0.75 (95% CI: 0.70, 0.80) on both cross-validation and holdout test.

**Figure 3:**
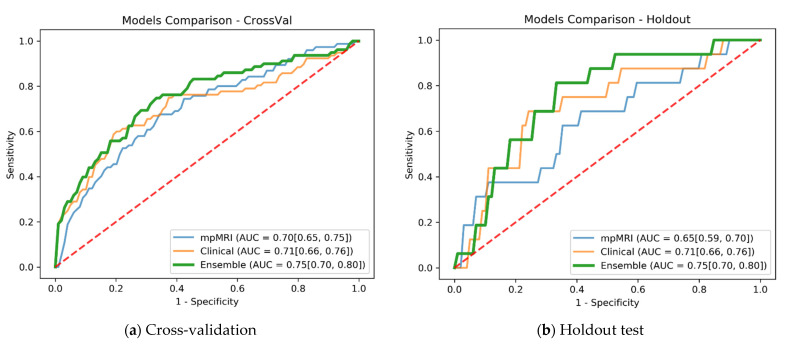
Cross-validation and holdout ROC curves for the mpMRI model, clinical model, and final ensemble model. (**a**) Cross-validation evaluation with ensemble model AUC of 0.75 (95% CI: 0.70, 0.80). (**b**) Holdout evaluation with ensemble model AUC of 0.75 (95% CI: 0.70, 0.80).

Figure 3 compares the ROC curves of the mpMRI, clinical, and final ensemble models in cross-validation and in holdout independent test. The ROC curves exhibit significant trends regarding specificity at several sensitivity operation points. In both cross-validation and holdout, the mpMRI model shows promise in predicting recurrence with good specificity at 0.9 sensitivity operation points. The clinical model demonstrates the capacity to predict recurrence with high specificity around the 0.6 sensitivity operation point, but lower specificity in the high-sensitivity operation points. The ensemble of mpMRI and clinical data leveraged both modalities and improved the AUC and overall specificity at various operation points.

To further evaluate and compare the models, we selected several high-sensitivity operation points and calculated specificity, F1-score, balanced accuracy, PPV, and NPV at these points. High-sensitivity operation points are deployed in clinical practice and thus are of special interest. Choosing a high-sensitivity operation point in our problem setting means that almost all patients that suffered recurrence are correctly classified by our model. Early accurate prediction of these patients can enable their treatment options to be reassessed in advance, reducing the risks of ineffective or unnecessary treatment.

Table 3 summarizes the results at sensitivity operation points 0.87, 0.90, and 0.93. Once again, the ensemble model performs better than the individual models in all metrics at high-sensitivity operation points. We obtained the same metrics’ values for a sensitivity of 0.87 and sensitivity of 0.90 in the holdout test due to the limited size of the holdout set.

We also used the McNemar test [12] to calculate the *p*-value when comparing the predicted labels of the individual models with those of the ensemble model at the selected operation points. On the McNemar test *p*-value, we applied Bonferroni correction for multiple hypotheses (α = 0.05, 12 tests, significance observed when *p*-value < 0.0042). The results are that in cross-validation, all comparisons in all the three sensitivity operation points are statistically significant, except when comparing mpMRI and the ensemble models at a sensitivity operation point of 0.87. For the holdout test, due to its limited number of patients, the only comparison that was statistically significant was the comparison of the clinical and ensemble models at a sensitivity operation point of 0.93.

### 3.2. Explainability

**Figure 4:**
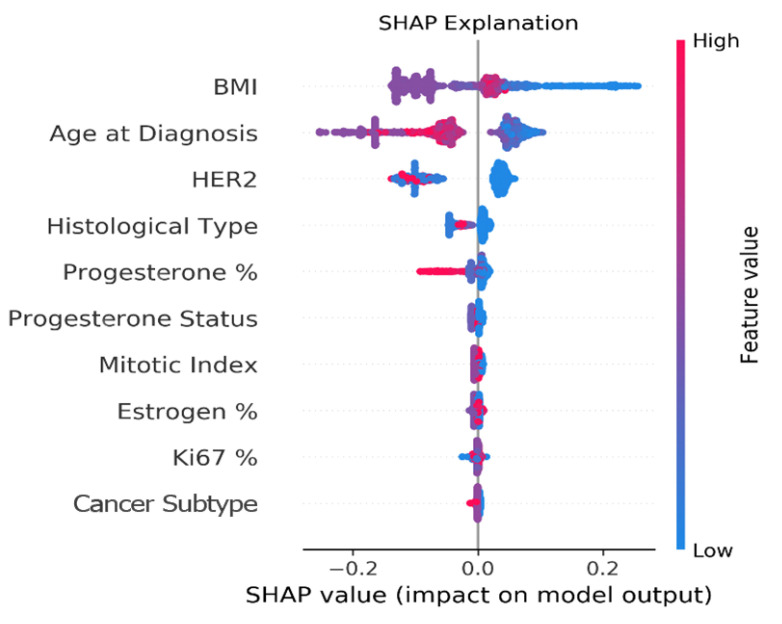
Clinical feature contributions. A summary plot of the SHAP values of top features in the clinical model. Each point represents a single patient. The *x*-axis indicates the effect (either positive or negative) of the feature on the predicted score for the patient. The point’s color represents the value of the features (red = high value, blue = low value, purple = close to the average value).

Figure 4 provides an explanation of the clinical model via the SHAP algorithm. SHAP considers all possible combinations of features with and without a specific feature to evaluate its contribution to the prediction. It reveals each feature’s importance and demonstrates how each feature of each patient affects the predictive model results. The figure depicts the top 10 clinical features in descending order that had the most influence on the five-year recurrence prediction. A positive SHAP value means a positive impact on the prediction, while a negative value leads the model to predict ‘recurrence-free’. The point’s color represents the values that each feature can take, including red for high values, blue for low values, and purple for values that are close to the average value.

The categorial clinical features in the data can take the following values: HER2: 0—HER2 negative, 1—HER2 positive; histological type: 1—NST, 2—lobular, 3—medullary, 4—other; progesterone status: 0—progesterone negative, 1—progesterone positive; mitotic index: number of mitoses; and cancer subtype: 1—TNBC, 2—LuminalA, 3—LuminalB, 4—HER2+.

Interestingly, BMI and age at diagnosis are ranked highest in terms of association with the outcome. In particular, lower values of BMI as well as younger age at diagnosis tend to have higher risk of five-year recurrence. To further analyze the robustness of these results and assess if the above feature ranking persists when we exclude patients with missing values, we checked the SHAP explanation on various subsets of the patients’ cohorts. Appendix C shows that training with subsets of the data, i.e., excluding patients with missing values, exhibits very similar SHAP rankings as the original clinical model trained on the entire Clinical cohort. These results are discussed in Section 5.

### 3.3. Subgroup Analysis

We divided each of our cohorts into prognostic subgroups following the clinicians’ guidance regarding features that characterize patients expected to have similar outcomes. We chose to define the different groups by leveraging the following five features: (1) age (≤50, 50–60, ≥60); (2) cancer subtype (luminal, TNBC, HER2+); (3) histological type (invasive carcinoma of no special type (NST), other); (4) tumor grade (I or II, III); and (5) Ki67 (below 15%, above 15%). In addition, each feature could have a missing value (no value). That is, we considered 432 (4 × 4 × 3 × 3 × 3) subgroups, and of those candidates, we only analyzed subgroups that contained at least 10 different patients with positive and negative outcomes within the subgroup. In the MRI+Clinical cohort, we analyzed 11 such subgroups, and in the Clinical cohort, we analyzed 26 such subgroups.

The analysis utilized the scores of the final ensemble model of the uncensored patients. For each subgroup, we computed the AUC with 95% CI via bootstrapping. Table 4, which is organized by the AUC scores, summarizes the subgroup analysis results for selected subgroups in which the AUC was above 0.85. Rows 1–2 outline the analysis of the MRI+Clinical cohort, in which patients have both clinical and MRI data. Rows 3–4 summarize the analysis of the Clinical cohort, in which patients have clinical data but only optionally have MRI data. We note that in some subgroups, the ensemble model can help differentiate patients that are in the same prognostic subgroup but have different outcomes. Our models can provide accurate prognosis (AUC > 0.89) for specific groups of young women with poor prognoses. Specifically, the subgroup with nonunanimous outcomes of women under 50 years of age that have luminal cancer, NST histological type, grade III tumor, and Ki67 above 15% is accurately predicted by our model in both cohorts, with increased accuracy from AUC 0.89 to AUC 0.94 when imaging is utilized.

## 4. Related Work

Our work focuses on the capacity to predict the recurrence of breast cancer based on MRI and clinical data. In this section, we review previous work that leveraged clinical history, MRI images themselves, or human interpretations of MRI images to predict recurrence within five years or a similar outcome.

Chen et al. [13] achieved impressive results with a multi-classifier multi-objective (MCMO) model outperforming various single classifiers by a clear margin. The model was applied to a cohort of 114 patients and recurrence within 36 months was predicted. The MCMO model is based on non-imaging features such as demography and histopathology. In a study by Tseng et al. [14], prediction of a three-month prognosis for metastasis is introduced. They used a random-forest-based model together with serum biomarkers and clinicopathological data from 144 patients, but no imaging data were used directly in the model. Note that in both papers, the outcomes are defined using a shorter time span than the one we used in our analysis. Naturally, that enables more accurate results.

There have been some approaches to utilizing MRI images to predict the recurrence of cancer. For example, tumor volume approximation [15,16] and texture features have been shown to have a clear connection to recurrence [17,18]. In another study [19], both morphological (rim enhancement, etc.) and quantitative parameters (entropy, kurtosis, etc.) were extracted from perfusion T1 MRI data using a semi-automatic approach, and a significant connection from some of these features to the prognosis was identified. Phenomena such as tumor rim enhancement [20], peritumoral edema [21], and background parenchymal enhancement [22] have also been identified to be related to a patient’s future outcome in previous studies and could be automatically extracted from MRI images.

Deep learning and convolutional neural networks have been shown to be effective in breast cancer studies when predicting pathological complete response (pCR) using MRI data. Liu et al. [23] successfully used a standard CNN model with manually segmented MRI data to distinguish 131 patients as pCR and non-pCR. Duanmu et al. [24] used a deep learning model without the need for segmentation and achieved good accuracy in the prediction of pCR on a curated subset of 112 patients from an I-SPY1 trial. The fusion of deep learning MRI features and clinical information was found very effective in predicting pCR in a study by Joo et al. [25]. In a study by Peng et al. [26], pCR was predicted, and the main finding was that the deep learning model clearly outperformed classical linear discriminant analysis. This motivated us to consider that recurrence could also be predicted better using neural networks.

Multiparametric MRI has been used before in breast cancer studies, for example, to classify pre-segmented lesions into benign and malignant [27]. In that study, T2 and DCE MRI data were used as input and all the features were generated with a CNN model. The work in [28] used multiparametric MRI, T2, and DCE to predict pathologic complete response to neoadjuvant chemotherapy. Similar to our approach, Comes et al. [29] predicted three-year breast cancer recurrence using CNN-generated imaging features combined with some clinical features. Instead of Dixon MRI used in our work, they based their analysis only on DCE-MRI data. Moreover, they also used imaging acquired after treatment start, while our work uses pre-treatment imaging only. To the best of our knowledge, there are no multiparametric MRI models for predicting recurrence. Moreover, the Dixon MRI sequence has rarely been used in breast cancer AI models.

Perhaps the closest counterpart in the literature to our study is the method known as RESONATE [30], where deep learning classification was combined successfully with numerical features extracted from the segmented tumor region. Whereas in that study only DCE volume was used, we use Dixon sequences and ADC volume computed from a diffusion-weighted MRI to calculate the volumetric features. Some of the features used in RESONATE are very similar to what we use in our work, and some of our features are novel. Our approach also includes an automatic segmentation method that detects the tumor region from other breast tissue.

Several online calculators give estimations for such outcomes as survival and recurrence probability. Perhaps the most relevant calculator is PredictBreast [31], but it applies only to patients who have already had a surgery, whereas our point of prediction is prior to surgery. Another good candidate is the CancerMath outcome calculator [32], but the outcome time span is incompatible with the survival data recorded in our dataset (15 years vs. 5 years). The same problem applies to the CTS5 calculator [33]. The Neoadjuvant Therapy Outcomes Calculator [34] calculates the anticipated five-year distant metastasis-free survival, but it requires a post-treatment pathologic stage, whereas our point of prediction is before treatment. Overall, we did not find an online outcome calculator that could be used in our research settings. Interestingly, all calculators except the latter use age as one of the features for their prediction, and age is also used in our model. However, none of the calculators use the BMI feature, which was found to be a prognostic biomarker in our model.

The 3D volumetric features use an approach similar to that of Thakran et al. [35], but instead of T1, T2, and PD-weighted DCE-MRI images, we used water- and fat-only contrasts from the Dixon series and ADC volume from the breast diffusion MRI series. Thakran et al. used data from 30 subjects and were able to achieve a good match between manual and automatic breast segmentation. MRI texture and morphological features were also used in [36] to predict recurrence, although Eun et al. used MRI sequences different from ours.

To summarize, different related studies focused on the clinical modality or the imaging modality, or a combination that is limited to very few clinical modalities. The closest lines of work that generate prediction algorithms based on clinical data and medical images, while combining state-of-the-art deep learning technologies with XGBoost, random forest, and logistic regression applied to clinical data, are the studies aimed at identifying cancer in breast screening exams [37,38,39]. The analysis in those studies is different from our work in three ways: the algorithm task is different (detecting cancer in screening versus predicting five-year breast cancer recurrence), the images are X-ray-based versus magnetic resonance images, and the previous studies did not leverage classic image processing algorithms.

## 5. Discussion

In this paper, we explore the prediction of future cancer recurrence in women with locally advanced breast cancer who are treated with NAC. We introduce a multimodal prediction model that is based on clinical data and breast mpMRI images taken prior to NAC treatment. Our results demonstrate the ability to predict recurrence prior to NAC treatment initiation using each modality alone, but the multimodal model offers better results. We used deep learning and image processing algorithms to analyze our mpMRI data and classical machine learning algorithms to analyze the clinical data. Using two branches enabled us to use the best method per modality and utilize the maximum available data for each data type. Furthermore, we note that comparing the results on the holdout independent unseen cohort with the results on the cross-validation shows that the performance is similar. This holds the promise that our models may be able to generalize to unseen but similar datasets.

High sensitivity is important in our problem setting, as treatment of patients with likely recurrence is different and more aggressive compared to those who are not likely to encounter recurrence. We showed that at high-sensitivity operation points, the model performance is improved when adding the MRI modality. However, the model suffers from the usual limitations with nonzero false negative cases where the model’s prediction is that the treatment is effective for the patient, while in fact, it is ineffective, and the patient encountered post-treatment cancer recurrence within five years.

We also analyzed associations in the data and corroborated risk factors known to be associated with prognosis. The ranked list, which was automatically generated from the data, includes at the top known prognosis biomarkers/factors such as BMI, age at diagnosis, and HER2 expression. Note that BMI and age at diagnosis remained top features even when we trained the models with subsets of the data after excluding patients with missing values. The age at diagnosis SHAP explanation shows that younger women tend to have a greater risk of five-year recurrence. This can be explained by the fact that younger women tend to have triple-negative disease and high-grade tumors, which are known to be more aggressive [40]. In addition, the SHAP explanation shows that lower BMI is associated with greater risk of five-year recurrence. This counter-intuitive result has some evidence in prior literature. While Jiralerspong and Goodwin [41] show that obesity is associated with increased risk of recurrence, they also find that this association is not valid in some cancer subtypes, e.g., ER-negative and TNBC. Moreover, Modi et al. [42] studied the obesity paradox and found that in advanced breast cancer, higher BMI is significantly associated with improved overall survival. Assi et al. [43] also found that low BMI is associated with increased risk of premenopausal breast cancer. Our study indicates that BMI might serve as a predictor for recurrence in groups of women that are similar to our study setting, i.e., women that are offered NAC treatment and who are typically young women with locally advanced breast cancer that prefer breast-conserving surgery.

The HER2 biomarker was the third-ranked factor in the SHAP explanation. This ranking indicates that the HER2-negative population, which includes the triple-negative patients, is mostly associated with increased risk for five-year recurrence. It also shows that patients with HER2-positive expression before neoadjuvant therapy have a better prognosis, which is consistent with findings in prior publications [44,45]. One possible explanation for this is the anti-HER2 targeted therapy that became part of the standard of care in recent years [46].

Because the data studied here were collected retrospectively from a health provider, we acknowledge that it is biased and noisy. MRI has no standardized protocol for scan acquisition and high variance of image resolution, voxel size, and image contrast dynamics, resulting in extensive variety in the MRI data. We selected special MRI preprocessing and neural networks to adjust for these limitations and improve the signal-to-noise ratio. This preprocessing was essential for the major contribution of the MRI modality to the five-year recurrence prediction.

Dixon sequence volumes were included in our study, as they have been reported as a promising option for future breast imaging approaches [47,48]. The Dixon series also had other benefits. First, Dixon volumes visualize different tissues on the breast very efficiently, which eliminates the need for data annotation of these volumes. Second, the Dixon fat-only and water-only volumes do not need alignment as they are acquired in the same session. The same reason also supports the selection of the ADC volumes, as they can also be used without a significant alignment process with Dixon volumes due to the lower resolution of ADC volumes. In comparison with DCE-MRI, these selected methods are also non-invasive in nature, as they do not require the administration of contrast agent injections to patients. DCE-MRI was also found to be more challenging on voxel-level 3D segmentation, as it includes slight movements between 3D volumes in time due to the long measurement time of the DCE-MRI session. Most interesting is the improvement achieved by leveraging the Dixon sequence, which is rarely used in the literature, although its capability to separate water and fat tissues is relevant to breast cancer studies. Additionally, the Dixon and ADC volumes produced explainable features.

We stratified the data based on characteristics that are expected to define patients with similar prognoses. We found that in some subgroups, our multimodal method can help differentiate patients who are in the same prognostic subgroup but have different outcomes. Specifically, the subgroup of women under 50 years of age with poor prognoses is accurately predicted by our model in both cohorts, and the accuracy is increased when imaging analysis is utilized. However, the small number of patients in each subgroup created wide confidence intervals per group. Further validation of the method with a larger number of patients and with additional independent cohorts is a direction for future work.

A version of the ensemble method reported in this paper was used in the international BMMR2 challenge to predict pathologic complete response for patients treated with NAC. In this competition, we retrospectively analyzed an independent dataset of 193 patients collected in the I-SPY2 multicenter clinical trial [49]. The ensemble model we employed analyzed multimodal breast mpMRI and clinical data using methods similar to those in this paper, except that in the mpMRI component we extracted radiomics features instead of deep learning features. This change was necessary as the I-SPY2 is a smaller dataset and overfits when using high-level deep learning features but generalizes well when using low-level radiomics features. Our method analyzed clinical data along with longitudinal DCE-MRI and DW-MRI imaging using the open source FuseMedML [50], a PyTorch-based deep learning framework for medical data. The model won second place (AUC 0.838), with only a marginal difference from first place (AUC 0.840), and was a standout from the other entries in the challenge.

## 6. Conclusions

Breast cancer is a dynamic disease, and making an accurate prognosis is challenging. We focus on the question of early prediction of five-year cancer recurrence in women with breast cancer who are treated with NAC. Accurately predicting the future outcome of a patient based on data available prior to treatment initiation could impact the treatment planning and selection. We have rich information collected from 1738 breast cancer patients. The data include clinical information for all patients and medical images for some of the patients. We introduce a multimodal prediction model that is based on clinical data and breast mpMRI images taken prior to NAC treatment. We compared the performance of models on different data elements and evaluated them by AUC as well as by specificity, F1-score, balanced accuracy, PPV, and NPV at clinically important sensitivity operation points. The results of cross-validation and unseen holdout test show that the multimodal ensemble model that leverages both the mpMRI and the clinical models offers improved results over the unimodal models. We then used interpretability methods to explain the model and identify important clinical features for predicting recurrence. Finally, we stratified the data based on characteristics that are expected to define patients with similar prognoses. We found that in some subgroups, the ensemble model can help differentiate patients that are in the same prognostic subgroup but have different outcomes.

Future work may add additional modalities to our multimodal approach, such as data of histopathology imaging and gene expression profiling. We also seek to use bigger cohorts from additional sites to increase our training data and have better generalization, toward the large-scale validation of our models.

## Figures and Tables

**Table 1:** Cohorts and the number of patients in the dataset.

	Total Number of Patients	Uncensored Patients
Clinical cohort	1627	928
MRI+Clinical cohort	463	317
Holdout cohort	100	62

**Table 2:** Evaluation of the models on cross-validation and holdout test. Best results are in bold.

	Model	Cross-Validation AUC [95% CI]	Holdout Test AUC [95% CI]
1	Subtraction-only MRI	0.67 [0.61, 0.72]	0.64 [0.60, 0.70]
2	Multiparametric MRI (mpMRI)	0.70 [0.65, 0.75]	0.65 [0.59, 0.70]
3	Clinical	0.71 [0.66, 0.76]	0.71 [0.66, 0.76]
4	Ensemble subtraction-only MRI and clinical	0.73 [0.68, 0.78]	0.73 [0.67, 0.78]
5	Ensemble multiparametric MRI and clinical (final model)	**0.75 [0.70, 0.80]**	**0.75 [0.70, 0.80]**

**Table 3:** Cross-validation and holdout test of the per-modality models and the ensemble model at sensitivity operation points 0.87, 0.90, and 0.93.

Cross-Validation			
Metric	mpMRI Sens = 0.87, 0.90, 0.93	Clinical Sens = 0.87, 0.90, 0.93	Ensemble Sens = 0.87, 0.90, 0.93
Specificity	0.31, 0.18, 0.15	0.26, 0.16, 0.14	0.39, 0.35, 0.24
F1-score	0.42, 0.39, 0.39	0.40, 0.39, 0.39	0.45, 0.45, 0.42
Balanced accuracy	0.59, 0.54, 0.54	0.56, 0.53, 0.53	0.63, 0.63, 0.59
PPV	0.28, 0.25, 0.25	0.26, 0.25, 0.25	0.30, 0.30, 0.27
NPV	0.88, 0.86, 0.88	0.86, 0.85, 0.87	0.91, 0.92, 0.92
**Holdout Test**			
**Metric**	**mpMRI** **Sens = 0.87, 0.90, 0.93**	**Clinical** **Sens = 0.87, 0.90, 0.93**	**Ensemble** **Sens = 0.87, 0.90, 0.93**
Specificity	0.26, 0.26, 0.20	0.46, 0.46, 0.17	0.57, 0.57, 0.48
F1-score	0.44, 0.44, 0.44	0.51, 0.51, 0.44	0.56, 0.56, 0.55
Balanced accuracy	0.57, 0.57, 0.57	0.67, 0.67, 0.56	0.72, 0.72, 0.71
PPV	0.29, 0.29, 0.29	0.36, 0.36, 0.28	0.41, 0.41, 0.39
NPV	0.86, 0.86, 0.90	0.91, 0.91, 0.89	0.93, 0.93, 0.96

**Table 4:** Subgroup analysis for both cohorts, the MRI+Clinical cohort and the Clinical cohort.

MRI+Clinical Cohort							
	# Positives # Negatives	Age	Cancer Subtype	Histological Type	Tumor Grade	Ki67	AUC
1	2 pos, 9 neg	≤50	Luminal	NST	III	Above 15%	0.94 [0.75, 1]
2	3 pos, 20 neg	≤50	No value	NST	III	Above 15%	0.92 [0.70, 1]
**Clinical Cohort**							
	**# Positives** **# Negatives**	**Age**	**Cancer** **Subtype**	**Histological** **Type**	**Tumor** **Grade**	**Ki67**	**AUC**
3	2 pos, 10 neg	≥60	No value	NST	III	Above 15%	0.90 [0.67, 1]
4	10 pos, 16 neg	≤50	Luminal	NST	III	Above 15%	0.89 [0.74, 1]

**Figure A1:**
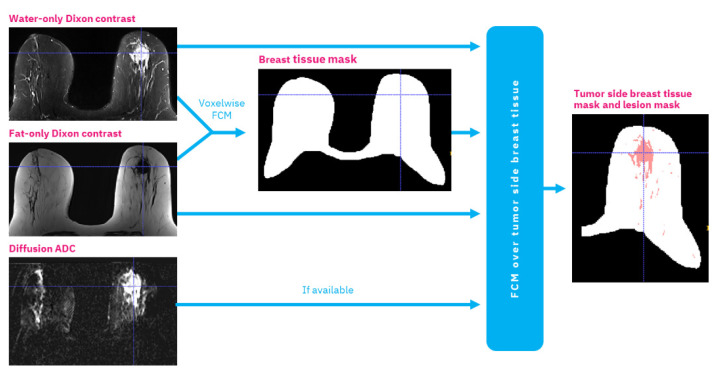
Breast tissue segmentation concept in the Dixon-ADC component. The first step with voxel-wise FCM (fuzzy C-means) segments the whole MRI volume to detect voxels, which belong to breast tissue. This is done with water-only and fat-only Dixon volumes. Then voxels that belong to the tumor side of the mask are again clustered with FCM using the same Dixon volumes, as well as diffusion ADC volume if it is available from the patient. The clusters with the biggest overlap on high-intensity areas on ADC and water volumes are selected as lesion regions.

**Figure A2:**
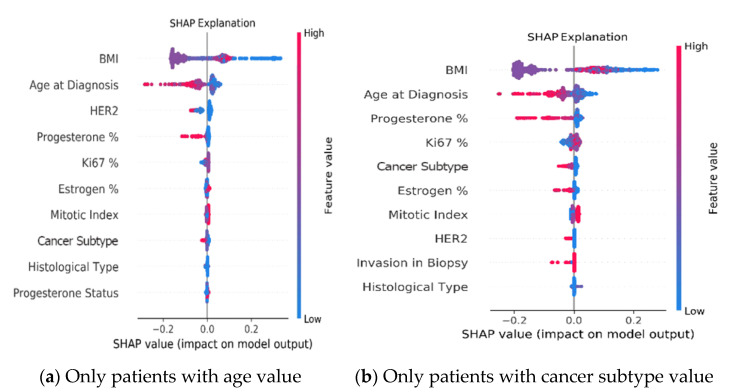
Clinical feature contribution with subsets of training data. (**a**) Training with only the patients that have age value. (**b**) Training with only the patients that have cancer subtype value.

## Data Availability

Data sharing is not applicable. However, as part of the BMMR2 challenge, a version of our method was used on the public dataset I-SPY2 from The Cancer Imaging Archive (TCIA): https://wiki.cancerimagingarchive.net/pages/viewpage.action?pageId=50135447 (accessed on 10 June 2022).

## Appendix A. Data Acquisition

We gathered a collection of data from a cohort of 1738 patients treated with NAC at Institut Curie. All the data were previously anonymized complying with all the necessary regulatory laws, making it impossible to identify the patients. Aside from the MRI images, a group of clinicians and other experts carefully designed a list of relevant clinical features to fully describe the state of the patient, the evolution of the cancer, and the outcome after NAC treatment. This collection of data comes from a manually curated database, and it includes the following items:

- Demographic data:
  ◦ Weight;
  ◦ Height;
  ◦ Age (in days) at time of diagnosis.
- Tumor data:
  ◦ Side of the tumor (right or left breast);
  ◦ Tumor number;
  ◦ Carcinoma in situ in the biopsy;
  ◦ Histological type (NST/ductal, lobular, medullary, other);
  ◦ Grade of the tumor on the Ellis–Elston (EE) scale;
  ◦ Ki67 percentage;
  ◦ Status of estrogen and progesterone receptors;
  ◦ Status of HER2;
  ◦ Type of cancer (TNBC, luminal A/B, HER2+).
- Treatment outcome data:
  ◦ Response to treatment, RCB (Residual Cancer Burden) scale;
  ◦ Response to treatment, Chevalier scale;
  ◦ Response to treatment, Sataloff scale;
  ◦ Postsurgical pathological T and N;
  ◦ Last news status (alive, dead) (if dead, age at death in days);
  ◦ Last news age (in days);
  ◦ Presence/absence of local relapse (+ age in days);
  ◦ Presence/absence of metastasis (+ age in days);
  ◦ Whether the patient died within five years after treatment.

Some of the features have missing values, so they could not contribute much to our models. Table A1 below describes the general characteristics of key features in the study population.

**Table A1:** Key feature characteristics in the dataset.

Feature	Total Patients with Value	Missing %
Age at diagnosis	1504	13.46%
EE grade	1467	15.59%
Histological type	1437	17.32%
Progesterone status	1419	18.35%
Estrogen status	1416	18.53%
Weight	1215	30.09%
HER2 positive	1169	32.74%
Mitotic index	1080	37.86%
Ki67 percentage	1051	39.53%
Height	893	48.62%
Cancer subtype	797	53.82%

To comply with regulations such as GDPR, the data analysis was performed using the model-to-data paradigm [51], where all the data remained within the Institut Curie infrastructure. The algorithms were transferred to a strong GPU-enabled server residing inside Institut Curie. The data analysis, training, and evaluation of the models were then carried out on that server.

## Appendix B. Dixon-ADC Segmentation

The Dixon-ADC component in the mpMRI is based on fuzzy c-means (FCM) clustering for automatic image segmentation with two phases: breast segmentation and lesion segmentation, generating three 3D masks: bilateral breast tissue mask, tumor side breast tissue mask, and lesion mask. The figure below (Figure A1) shows an example of such segmentation.

## Appendix C. Feature Explanations

The figure below (Figure A2) depicts SHAP explanations when excluding patients with missing values. We examined training the clinical model with just the 1504 patients who have age value or training with just the 797 patients who have the cancer subtype value. The figure shows the SHAP explanations generated for the clinical model in each case. We see that it exhibits the same important features and characteristics as the original clinical model trained on the entire Clinical cohort with BMI and age at diagnosis as the top features.
